# Assessment of skin response in T4b breast carcinoma patients post-neoadjuvant chemotherapy

**DOI:** 10.3332/ecancer.2021.1271

**Published:** 2021-07-28

**Authors:** Abhishek Sharma, Shagun Mahajan, Sanjit Kumar Agrawal, Rosina Ahmed, Debdeep Dey

**Affiliations:** 1 Department of Breast Oncosurgery, Tata Medical Center, Kolkata 700156, India; 2 Department of Pathology, Tata Medical Center, Kolkata 700156, India

**Keywords:** T4b, skin response, NACT, breast cancer

## Abstract

**Background:**

Breast cancer patients with skin ulcerations, satellite nodules or Peau d’orange at presentation are classified with stage 4 breast cancer (T4b). Neoadjuvant chemotherapy (NACT), followed by mastectomy, is the commonly accepted treatment in such patients for fear of adverse outcomes with breast conservation surgery (BCS) and uncertainty over sparing initially involved skin irrespective of the response to chemotherapy. Identifying patients with skin resolution post-NACT can help surgeons in decision-making.

**Aim:**

To assess skin response in T4b breast cancer patients post-NACT and find the correlation between various clinical and pathological factors associated with no skin involvement on final histology.

**Methodology:**

Records of breast cancer patients managed at the Tata Medical Center, Kolkata, with NACT for T4b breast carcinoma patients who underwent mastectomy were reviewed between January 2014 and December 2018. Final histology was checked for dermal involvement with the tumour. The Mann–Whitney U test was used for continuous variables for descriptive data, and Pearson’s chi-squared and Fischer’s exact tests were applied for categorical data. *p*-value < 0.05 was taken as significant.

**Results:**

A total of 285 records mentioning skin involvement were reviewed, out of which 111 patients fulfilled the AJCC criterion. The median age at diagnosis of T4b breast cancer was 50 years. The median clinical size pre-chemotherapy was 7 cm. Residual median tumour size on final histology was reported as 1 cm. 78/111 patients showed a post-NACT response of 50% or more, and 43/111 showed a response of more than 90%. 57 (51.4%) patients showed skin involvement on final histopathology, while 54 (48.6%) patients did not.

ER negative tumours were more likely to show no dermal involvement (*p* = 0.006). Residual tumour size of less than 1 cm on final histology (*p* < 0.05) and nodal stage were significant predictors of dermal response.

**Conclusion:**

Approximately half of the T4b breast cancer patients showed resolution of dermal skin involvement post-NACT. ER negative and those with residual tumour size less than 1 cm post-NACT are more likely to show dermal resolution. This can help surgeons plan a BCS or skin sparing mastectomy for such patients who usually end up having a mastectomy.

## Introduction

Breast cancer is the most common cancer among women. Approximately 2.1 million new cases were estimated to occur worldwide in 2018 [1]. Breast cancer lesions with oedema (including Peau d’orange), satellite nodules confined to the same breast and ulceration of skin are classified as T4b, which is essentially a clinical classification [2].

Very few studies of patients with locally advanced breast cancer (LABC) who underwent neoadjuvant chemotherapy (NACT) and conservative surgery exist, but they have shown breast-conserving surgery as a safe and effective therapy for carefully selected patients [3, 4].

In developing countries like India, a big chunk of the patients present with LABC, many of whom also have skin involvement or the tumour is quite close to the skin [5]. The management of these lesions is controversial. Traditionally, they have been placed in the highest T subgroup (T4), but many researchers have questioned this approach.

T4b breast cancer is poorly described in terms of its biological behaviour or its response to NACT. Furthermore, most of the few attempts to study T4 disease tend to club inflammatory breast cancer skin changes with non-inflammatory breast cancer, even though the two are different clinical entities [6–10].

T4b lesions undergo NACT, but an overwhelming majority of patients who present with clinical T4b are still treated with a mastectomy after NACT because it is ‘assumed’ that the local failure rates with breast preservation would be unacceptably high [6]. But this may not be true. Multiple studies have shown that breast conservation surgery (BCS) is safe and feasible in appropriately selected T4b patients. However, even if surgeons attempt BCS, there is an inclination to include the initially involved skin in the BCS specimen, thereby reducing the aesthetic outcome of BCS. This decision is sometimes taken without considering the post-NACT response.

To the best of our knowledge, there is a paucity of research to assess the skin response after NACT in T4b. It is, however, an important question. Does the tumour regress from the skin after NACT and are there any predictors of response? The answer to this question will help surgeons make informed decisions when choosing to do BCS or skin sparing mastectomy (SSM) in appropriately selected T4b breast cancer patients.

## Methodology

This was a single institute retrospective cohort study. Data were retrieved from prospectively maintained institutional Redcap database and clinical records. The study was approved by the institutional ethics committee with waiver number EC/WV/TMC/46/20.

Inclusion criteria encompassed all patients fulfilling the American Joint Committee on Cancer (AJCC) criterion of T4b, namely satellite nodules on the ipsilateral side, ulceration, Peau d’orange and oedema as judged by the treating surgeon on the first visit and who subsequently underwent mastectomy post-NACT. Patients who received less than four cycles of anthracycline and/or taxane-based chemotherapy, those who had more than 12 weeks gap from the end of chemotherapy to surgery and those who progressed or developed metastatic breast cancer were excluded from the study. Patients clinically labelled as skin free, but had mammographic evidence of skin involvement, were also not included in this study in keeping with the AJCC criterion [2].

A total of 285 consecutive patients with documented clinical skin involvement between January 2014 and December 2018 were screened for study eligibility criteria. Interestingly, the AJCC classification was not always followed when labelling ‘skin involved’ in the clinical record. Of the 285 patients, only 111 fulfilled the AJCC criterion for T4b. We mention this to highlight that T4b disease is poorly understood or classified even in tertiary cancer centres.

The standard NC regimen consisted of four cycles of AC (Doxorubicin [Adriamycin] 60 mg/m^2^ + Cyclophosphamide 600 mg/m^2^), followed by 4 or 12 cycles of T (Taxol® [paclitaxel] 175 mg/m^2^ or 80 mg/m^2^, respectively). Only few HER2-positive (7/42) patients received Trastuzumab in addition to AC-T.

All patients with T4b disease underwent mastectomy with axillary dissection within a median of 3 weeks after completion of NACT as per institutional protocol. The final histopathology report was noted for residual skin involvement. Dermis involved and/or dermal lymphovascular invasion was taken as the marker for residual skin involvement.

### Statistical analysis

Data normalcy was checked by the Shapiro–Wilk test. For descriptive data, Mann–Whitney U test was used for continuous variable, and Pearson’s chi-squared/Fisher’s exact tests were applied for categorical data. Multivariate analysis was carried out by using logistic regression. *p*-value < 0.05 was taken as significant. All analyses were carried out with the Statistical Package for the Social Sciences statistical software package, version 25.

## Results

### Patient characteristics

The median age at diagnosis of T4b breast cancer was 50 years. 62.2% of the patients were postmenopausal. Most patients underwent surgery within a month of completing chemotherapy. 85.6% of the patients received AC-T regimen and 6.3% patients received Trastuzumab along with anthracycline and taxanes. The median time between completion of NACT and surgery was 29 days.

### Tumour characteristics

The median clinical sizes pre-chemotherapy were 7 and 3 cm post-chemo. The residual median tumour size on final histology was reported as 1.6 cm. 64.9% (*n* = 72) of the patients had grade three tumour. The majority of the patients were oestrogen receptor-positive (57.7%) and progesterone receptor-negative (54.1%). 42 (37.8%) patients were HER2 receptor-positive. Only 6.4% (7/42) of HER2+ patients received anti-HER2 therapy.

Table 1a and b summarise the demographics of the study population.

### Variables of response

Overall, 57 (51.4%) patients showed skin involvement (dermal involvement) on final histopathology report and 54 (48.6%) patients did not show skin involvement. Only 10 (9%) patients showed a pathological complete response (PCR), but 19 patients (17%) had complete response in breast. 78/111(70.27%) patients showed a post-NACT response of 50% or more regression in tumour size, while 43/111(38.73%) showed a response of 90%.

The dermal response had a significant association with hormone receptor positivity. Patients with ER-positive tumours were less likely to have dermal regressions (40/64) than ER-negative tumours (26/47) (*p* = 0.006). A similar association was also noted with PR negativity (*p* = 0.001). The number of patients who were HER2+ and received anti-HER2+ therapy was too small and, therefore, not subjected to statistical analysis.

Table 2 Correlation between clinicopathological variables and skin (dermis) involvement. A significant *p*-value is seen with ER-negative tumours and PR-negative tumours. Residual tumour size on final histopathology and N stage also showed significant *p*-values.

The dermal response was independent of age, initial tumour size or grade of tumour. It was also independent of BMI or menopausal status. The size of the residual tumour on histology was also significant. Patients with <1 cm tumour size on final histology were more likely to have dermal regression (*p* < 0.001). Out of the 40 patients with residual size <1 cm, 32 (80%) patients had no dermal involvement, while out of the 71 patients who had >1 cm residual tumour involvement, 49 (69%) patients had dermal involvement (*χ*^2^ = 24.6, *p* < 0.01).

## Discussion

Breast tumours having significant skin involvement have been classified as T4b since the first edition of the AJCC staging system, published in 1977 [7]. Management of T4b lesions is complex and challenging [8, 9]. Various authors have questioned placing these lesions into the highest T category only because of skin involvement [10, 11]. Once a lesion is placed in the T4 category, it becomes ‘unfit’ for breast conservation. This is clearly visible in the bias shown towards these lesions by excluding them from most large studies on BCS [12–14] after NACT.

Silverman *et al* [11] have shown that T4b lesions have the same prognosis as their counterparts of identical size and nodal status if skin involvement was ignored. They have called for treating these lesions based on their size and biology and to not be alarmed by their T4 status. Guth *et al* [10] have called placing lesions with skin involvement into T4 category ‘a historical mistake’. Their logic that skin involvement may merely be a coincidence of a smaller lesion which originated near to skin envelope certainly finds merit.

This brings us to question as to why these tumours usually get denied BCS or SSM after NACT. It is conveniently assumed that even if T4b lesions showed good clinical response to NACT, they should still undergo mastectomy because of high failure rates [9]. Large trials that have showed BCS efficacy after NACT in ‘large operable tumours’ used a cut-off of 3 or 5 cm tumours but excluded T4b lesions from their study population [12–14]. Hence, there is a paucity of data to decide for or against this logic. In fact, there is a lack of data to assess how non-inflammatory skin involved tumours behave after NACT. It is now generally accepted in post-NACT patients that ‘neomargins’ during surgery should be based on residual tumour and not the original tumour. The same logic could be extrapolated to skin response.

Our data show that in T4b breast cancer, the PCR was 17% in the breast and overall PCR (breast + axilla) was 9%. It is in accordance with similar findings from other studies around the world. Shen *et al* ([6)] reported a PCR of 24% in a selected group of T4b patients. McIntosh *et al* [14] from Aberdeen breast clinic demonstrated a 17% complete clinical response in a subset of T4b breast cancers [14]. We also report that 78/111 (70.27%) patients showed a post-NACT response of 50% or more and 43/111(38.73%) showed a response of 90% or more. This is despite the fact only 7/42 patients received Trastuzumab in our study, mainly due to financial constraints. If Trastuzumab were added to the chemotherapy, the above figures are expected to improve. All these patients are candidates for BCS or SSM and can avoid mastectomy.

Our study also visits the perennial question of what is a T4b tumour. Historically, any skin change has been considered T4b, and clinicians have been guilty of not adhering to AJCC classification. In our study, only 111 out of 285 (38%) patients initially classified as skin involved actually fulfilled the AJCC criterion. Many of those not fulfilling the AJCC criterion had residual dermal involvement on the final histopathological examination (HPE). Other studies have also described this problem. In an international survey, 70% of clinicians wrongly identified the criteria for T4b disease, leading the authors to caution the interpretation of data (1990–2000) of the T4b subset [15]. In fact, it was only the 2001 supplement of Tumour Node Metastasis (TNM) classification that clarified that the histological involvement in the absence of classical clinical signs does not constitute T4b [16].

The median tumour size of T4b tumours in our series was 7 cm (range = 2–15 cm). Some of the other series have reported a similar median tumour size [6, 17, 19]. The dermal response was independent of initial tumour size, grade or menopausal status. It was also not dependent on BMI, duration of chemotherapy or multifocality of the tumour.

In our series, women with ER positivity were less likely to have a dermal response. While 70% of ER-positive patients had dermal involvement after NACT, only 29% of ER-negative patients had dermal involvement (*p* = <0.01). This is in accordance with the generally observed findings of ER-negative tumours showing better response to NACT. Ring *et al* [18] showed similar findings: patients with ER-negative tumours were more likely to achieve a PCR than patients who were ER-positive (21.6 versus 8.1%, *p* < 0.001) [20].

Significant correlation was found between patients whose residual tumour size was less than 1 cm and no dermal involvement (Table 3). Out of the 40 patients with a residual size ≤ 1 cm, 32 (80%) patients had no dermal involvement, while out of the 71 patients who had more than 1 cm residual tumour involvement, 49 (69%) patients had dermal involvement (χ^2^ = 24.6, *p* < 0.001). This is similar to findings by Shen *et al* [6], who in a cohort of 33 patients with T4b disease identified that up to 88% of the patients had no dermal involvement in final pathology. Cance *et al* [20] identified post-treatment tumour size of less than 1 cm as an independent prognostic factor for locoregional relapse (HR = 1.04; 95% CI = 1.01–1.05, *p* = 0.01).

There has been a historical bias in excluding T4b lesions from BCS for the fear of higher locoregional recurrence (LRR). However, in the few trials that have included these patients (Table 4), LRR rates have been comparable to other large breast cancers. Some studies have shown BCS to have lesser LRR than mastectomy [14]. Overall, BCS has acceptable outcomes in patient with good clinical response post-NACT in T4b lesions [6, 17, 19, 20].

The important question when planning BCS or SSM for T4b lesions post-NACT is whether it is safe to spare the skin post-NACT when clinical skin resolution has occurred. Our study shows that more than half of the patients achieve resolution of dermal involvement following NACT. This proportion is higher if the residual tumour is less than 1 cm in size or the patient is ER-negative.

When doing BCS the surgeon needs to decide whether the skin initially involved but showing clinical resolution can be spared or should be included in the surgical specimen. The authors hope the above-mentioned data will help in decision-making.

This study has many limitations. This is a retrospective study which does not allow inferring whether microscopic resolution of dermal involvement benefits in corresponding increase in disease-free survival (DFS) or overall survival (OS). This question can conclusively only be answered by a randomised controlled trial (RCT).

Histologically speaking, breast cancer is staged as T4b when the invasive carcinoma directly invades into the dermis or epidermis with skin ulceration. Microscopically, the tumour has to infiltrate and ulcerate the epidermis to be classified as T4b. Cases in which there is ulceration of the skin only without any infiltration of the epidermis by tumour will not be considered as T4b. Therefore, cases which grossly mimic skin involvement very often present as dermal involvement without any skin ulceration, thereby not strictly complying with the T4b criteria according to AJCC.

**Figure 1.**
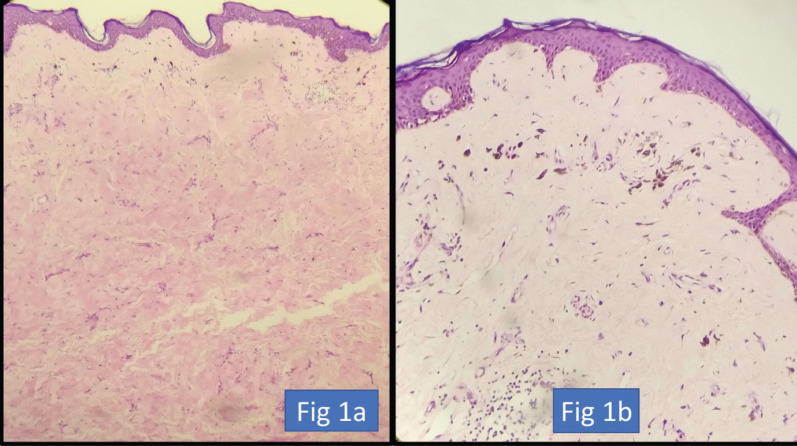
(a) HE ×200. Atrophied epidermis with loss of adnexal structures. Dermis showing hyalinisation and fibrosis indicative of treatment-related changes. (b) HE ×400. Dermis showing fibrosis, chronic inflammation and deposition of pigment-laden macrophages.

We have included no dermal involvement post-NACT as skin resolution (Figure 1). The AJCC criterion mentions only epidermis involvement as ypT4. If we take post-NACT AJCC criterion as skin involvement, then only 11 patients did not have skin resolution; however, that information is rather not helpful as a surgeon cannot leave behind skin where the dermis is still involved.

We have very few patients (7/42) in this subset who received anti-HER2 treatment, mainly due to financial constraints. We believe the response rates will go up if these patients were to receive Trastuzumab.

We have not classified the tumours on the present biological classification of Luminal A, B, HER2-enriched and triple negative, mainly because the HER2-enriched tumours have not been treated with targeted therapy. As targeted therapy has become more affordable in our country, we hope to provide this data in future.

## Conclusion

Approximately half of T4b breast cancer shows resolution of dermal skin involvement post-NACT. ER-negative and those with residual tumour size less than 1 cm post-NACT are more likely to show dermal resolution. This can help surgeons plan a BCS or SSM for such patients who usually end up having a mastectomy.

## Abbreviations

Neoadjuvant chemotherapy (NACT), Locally advanced breast cancer (LABC), Breast conservation surgery (BCS), Locoregional recurrence (LRR)

## Conflicts of interest

The authors declare that they have no conflicts of interest.

## Funding

The authors declare that no funding was received for the study.

## Figures and Tables

**Table 1(a).** Demographics of the study population.

Parameters	Median (range)
Age (years)	50 (23–73)
Time between chemotherapy and surgery (Days)	29 (20–180)
cT size before chemotherapy (cm)	7 (2–15)
cT size post-chemotherapy (cm)	3 (0–9)
cT ratio before and after chemotherapy	0.5 (0–1)
pT size (cm)	1.6 (0–13.3)

**Table 1(b).** Demographics of the study population.

Parameters (*n* = 111)	*n* (percentage)
Body mass index	
Underweight	5 (4.5)
Normal	34 (30.6)
Overweight	42 (37.9)
Obese	30 (27)
Menopausal status	
Premenopausal	42 (37.8)
Postmenopausal	69 (62.2)
NACT regimen	
AC-T	95 (85.6)
AC	9 (8.1)
AC-TH	7 (6.3)
Skin involvement on final histology	
Involved	57 (51.4)
Not involved	54 (48.6)
Grade	
1	2 (1.8)
2	37 (33.3)
3	72 (64.9)
Oestrogen receptor status	
Positive	64 (57.6)
Negative	47 (42.4)
Progesterone receptor status	
Positive	51 (46)
Negative	60 (54)
HER2 receptor	
Negative	47 (42.3)
Equivocal	22 (19.8)
Positive( without Trastuzumab)	35 (31.5)
Positive ( with Trastuzumab)	7 (6.4)

**Table 2.** Demographics of the study population (continued).

Variable	Total (*n* = 111)	Skin (dermis) not involved on final histology n (%)	Skin (dermis) involved on final histology n (%)	*χ* ^2^	*p*-value
Body mass index (BMI)					0.971 (Fisher’s exact)
Underweight	5	2 (3.7)	3 (5.3)		
Normal	34	16 (29.6)	18 (31.6)		
Overweight	42	21 (38.9)	21 (36.8)		
Obese	30	15 (27.8)	15 (26.3)		
Menopause (*n* = 283)				1.95	0.16
Premenopausal	42	24 (44.4)	18 (31.6)		
Postmenopausal	69	30 (55.6)	39 (68.4)		
NACT regimen				3.69	0.15
AC-T	95	43 (79.6)	52 (91.2)		
AC	9	7 (13)	2 (3.5)		
AC-TH	7	4 (7.4)	3 (5.3)		
Grade				0.66	0.71
1	2	1 (1.9)	1 (1.8)		
2	37	20 (37)	17 (29.8)		
3	72	33 (61.1)	39 (68.4)		
Oestrogen receptor status				7.52	<0.01
Positive	64	24 (44.4)	40 (70.2)		
Negative	47	30 (55.6)	17 (29.8)		
Progesterone receptor status				11.27	<0.01
Positive	51	16 (29.6)	35 (61.4)		
Negative	60	38 (70.4)	22 (38.6)		
HER2 receptor status				Not analysed due to small sample size	
Positive	35	24 (44.4)	11 (19.3)		
negative	47	21 (38.9)	26 (45.6)		
Equivocal	22	6 (11.1)	16 (28.1)		
Positive and Trastuzumab received	7	3 (5.6)	4 (7)		
Residual tumour size on final histology				24.60	< 0.01
Less than 1 cm	40	32 (59.3)	8 (14)		
More than 1 cm	71	22 (40.7)	49 (86)		
Multifocality				0.25	0.61
No	84	42 (77.8)	42 (73.7)		
Yes	27	12 (22.2)	15 (26.3)		
*N* stage on final histology				19.80	<0.01
0	33	26 (48.1)	7 (12.3)		
1	29	13 (24.1)	16 (28.1)		
2	23	9 (16.7)	14 (24.6)		
3	26	6 (11.1)	20 (35.1)		

**Table 3.** Univariate analysis of clinicopathological variables and skin (dermis) involvement.

Variable	U-value (Mann–Whitney)	*p*-value
Age	8,551.500	0.187
Time between chemotherapy and surgery	8,917.500	0.720
Clinical size of tumour before chemotherapy	8,586.500	0.227
Clinical size of tumour post-chemotherapy	4,596.000	<0.01
Ratio of clinical size of tumour before chemotherapy and clinical size of tumour post-chemotherapy	4,895.500	<0.01
Residual tumour size on final histology	5,808	<0.01

**Table 4.** Comparison of data of some of the other major trials who have reported on BCS in T4b patients.

Study	Inclusion criteria	Total patients	T4b patients	Tumour size	PCR	Skin involvement	LRRat 5 years in BCS
Shen *et al* [6]	T4b with good response	33	33	7 cm (2–12 cm)	Breast 30%	4 (12%)	15%
Carrara *et al* [17]	Stage 3 operable	449	175	7.15+−2.15dp	77 (17.1%) breast	–	6%
McIntosh *et al* [14]	Tumour greater than 4 cm	173	36	–	15%	–	2%
Touboul *et al* [19]	Stage 3 and stage 4	137	34	6 cm (1–16 cm)	9% overall	–	16
Cance *et al* [20]	Stage 2 and stage 3	59	17	–	15%	–	10%
Present study	All T4b	111	111	7 cm (2–15 cm)	9% overall, breast (17%)	51%	n/a
